# POLQ Overexpression Is Associated with an Increased Somatic Mutation Load and PLK4 Overexpression in Lung Adenocarcinoma

**DOI:** 10.3390/cancers11050722

**Published:** 2019-05-24

**Authors:** Kazuya Shinmura, Hisami Kato, Yuichi Kawanishi, Katsuhiro Yoshimura, Kazuo Tsuchiya, Yoshiyuki Takahara, Seiji Hosokawa, Akikazu Kawase, Kazuhito Funai, Haruhiko Sugimura

**Affiliations:** 1 Department of Tumor Pathology, Hamamatsu University School of Medicine, Hamamatsu, Shizuoka 431-3192, Japan; hisami@hama-med.ac.jp (H.K.); ky@hama-med.ac.jp (K.Y.); tsuchika@hama-med.ac.jp (K.T.); A14058@hama-med.ac.jp (Y.T.); hsugimur@hama-med.ac.jp (H.S.); 2 Advanced Research Facilities and Services, Preeminent Medical Photonics Education and Research Center, Hamamatsu University School of Medicine, Hamamatsu, Shizuoka 431-3192, Japan; yuichika@biglobe.jp; 3 Department of Otolaryngology/Head and Neck Surgery, Hamamatsu University School of Medicine, Hamamatsu, Shizuoka 431-3192, Japan; seijih1969@gmail.com; 4 Department of Surgery 1, Hamamatsu University School of Medicine, Hamamatsu, Shizuoka 431-3192, Japan; akawase@hama-med.ac.jp (A.K.); kfunai@hama-med.ac.jp (K.F.)

**Keywords:** lung adenocarcinoma, POLQ overexpression, PLK4 overexpression, somatic mutation load, centrosome amplification, translesion DNA synthesis

## Abstract

DNA Polymerase Theta (POLQ) is a DNA polymerase involved in error-prone translesion DNA synthesis (TLS) and error-prone repair of DNA double-strand breaks (DSBs). In the present study, we examined whether abnormal POLQ expression may be involved in the pathogenesis of lung adenocarcinoma (LAC). First, we found overexpression of POLQ at both the mRNA and protein levels in LAC, using data from the Cancer Genome Atlas (TCGA) database and by immunohistochemical analysis of our LAC series. POLQ overexpression was associated with an advanced pathologic stage and an increased total number of somatic mutations in LAC. When H1299 human lung cancer cell clones overexpressing POLQ were established and examined, the clones showed resistance to a DSB-inducing chemical in the clonogenic assay and an increased frequency of mutations in the *supF* forward mutation assay. Further analysis revealed that POLQ overexpression was also positively correlated with Polo Like Kinase 4 (PLK4) overexpression in LAC, and that PLK4 overexpression in the POLQ-overexpressing H1299 cells induced centrosome amplification. Finally, analysis of the TCGA data revealed that POLQ overexpression was associated with an increased somatic mutation load and PLK4 overexpression in diverse human cancers; on the other hand, overexpressions of nine TLS polymerases other than POLQ were associated with an increased somatic mutation load at a much lower frequency. Thus, POLQ overexpression is associated with advanced pathologic stage, increased somatic mutation load, and PLK4 overexpression, the last inducing centrosome amplification, in LAC, suggesting that POLQ overexpression is involved in the pathogenesis of LAC.

## 1. Introduction

Lung cancer is the most commonly diagnosed cancer (11.6% of the total cases) and is the leading cause of cancer death (18.4% of the total cancer deaths) in both sexes, according to the global cancer statistics, GLOBOCAN 2018 [1]. There are several histopathological types of lung cancer, and although the predominant histopathological type varies depending on the ethnic background, smoking status, and geographic location of the population, lung adenocarcinoma (LAC) is widely recognized as being one of the main histopathological subtypes [2,3]. Recent large-scale molecular characterization projects for LAC, such as the Cancer Genome Atlas (TCGA) project, have provided much information on the molecular characteristics of LAC, at the levels of DNA base alteration, DNA CpG methylation, mRNA expression, etc. [4,5,6,7]. However, since even such large-scale studies have not yet fully revealed the molecular characteristics of LAC and much still needs to be elucidated in this field, further basic and clinical studies on LAC are still being performed worldwide. Some such studies have utilized large-scale genomic alteration data provided by the above-mentioned projects [8]; since novel findings can be obtained by analyzing these data from different points of view, it is considered that there is a merit in utilizing them.

POLQ, also known as DNA polymerase theta (Polθ), is a DNA polymerase that is involved in processes such as translesion DNA synthesis (TLS) and DNA double-strand break (DSB) repair [9,10]. The C-terminal third of the POLQ protein is a family A DNA polymerase domain and the N-terminal third is a helicase-like domain with DNA-dependent ATPase activity [11,12]. TLS mediated by POLQ occurs at a low fidelity, therefore, POLQ is considered to be one of the error-prone DNA polymerases [12,13]. The DSB repair pathway involving POLQ is termed polymerase theta-mediated end-joining (TMEJ), also known as alternative non-homologous end-joining (alt-NHEJ) or alternative end-joining (altEJ), which is distinct from classical non-homologous end-joining (cNHEJ) repair initiated by the Ku proteins [14,15,16,17,18]. Both TMEJ and cNHEJ are more error-prone repair pathways as compared to homologous recombination, another DSB repair system [19]. Among human malignancies, POLQ mRNA overexpression has recently been reported in cancers of the stomach, colorectum, lung, breast, ovary, uterus, and oral cavity [17,20,21,22,23,24,25]. In regard to LAC, Kawamura et al. [20] reported low-level upregulation of POLQ mRNA expression in three out of 12 LACs (25%), while Allera-Moreau et al. [24] reported POLQ mRNA overexpression in 75 out of 93 LACs (81%), indicating that there are wide variations in the reported proportions of LACs showing POLQ overexpression. Moreover, analysis of POLQ expression at the protein level has been performed in only a few cases; POLQ protein expression was analyzed in only one of the seven studies referred to above [20], so that it still remains unclear whether POLQ protein is also overexpressed in cancers. Furthermore, as POLQ is an error-prone polymerase [9,10], it was speculated that POLQ overexpression could be associated with the mutation burden in LAC, however, there have been no reports to date on the correlation between the POLQ expression level and the number of somatic mutations in LAC. Therefore, the status of POLQ mRNA and protein expression and its relation to the number of somatic mutations in LAC still need to be determined.

In the present study, we hypothesized that abnormal POLQ expression might contribute to the pathogenesis of LAC via inducing somatic mutations and bestowing other characteristics, and investigated POLQ expression at the mRNA and protein levels and the relationship between POLQ expression and number of somatic mutations in LAC. Since POLQ overexpression was detected in LAC, the effects of POLQ overexpression in lung cancer cell line-derived clones were also investigated. Furthermore, POLQ overexpression was found to be concurrently associated with Polo Like Kinase 4 (PLK4) overexpression, which was also examined in the lung cancer cell clones. Finally, we investigated whether POLQ overexpression is observed in other types of human cancers.

## 2. Results

### 2.1. Overexpression of POLQ mRNA and Protein in LAC

**Figure 1:**
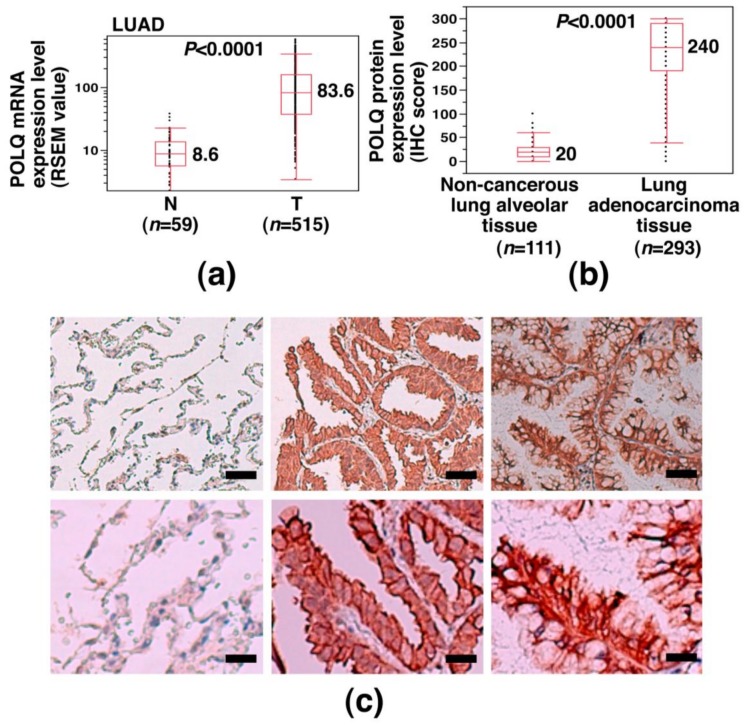
Overexpression of POLQ mRNA and protein in primary lung adenocarcinoma (LAC). (**a**) Detection of POLQ mRNA overexpression in LAC determined using data from the TCGA database (ID: LUAD). A Mann–Whitney *U* test was used for statistical comparison of the findings between non-cancerous tissue (N) and cancerous tissue (T); the *p*-value and median expression levels are shown. (**b**) Overexpression of POLQ protein in LAC determined by IHC analysis using rabbit anti-POLQ polyclonal antibody in cases of our hospital (HUH). A Mann–Whitney *U* test was used for statistical comparison of the findings between non-cancerous lung alveolar tissue and LAC tissue; the *p*-value and median expression levels are shown. (**c**) Representative IHC results of POLQ protein expression in primary LAC. The leftmost panel represents the results in non-cancerous lung tissue, while the remaining panels show the results in LAC tissue. The lower panels show a part of the upper panels at a higher magnification. Scale bar = 50 μm (upper); 20 μm (lower).

To determine the status of POLQ expression in LAC, we examined the POLQ mRNA expression level in LAC using the TCGA dataset. The results showed significantly higher POLQ mRNA expression levels in LAC tissue specimens than in non-cancerous lung tissue specimens (median expression value: 83.6 vs. 8.6; *p* < 0.0001) (Figure 1a) and POLQ overexpression was detected in 440 out of 515 cases of LAC (85.4%). We then investigated whether POLQ protein is also overexpressed in LAC. Immunohistochemical (IHC) analysis using an anti-POLQ antibody was performed in specimens collected from 293 patients with primary LAC at our hospital, and the results showed that POLQ protein, which was predominantly localized in the cytoplasm of the cells, was expressed at significantly higher levels in the LAC tissues than in the non-cancerous lung alveolar tissues (median H-score: 240 vs. 20; *p* < 0.0001) (Figure 1b,c). Moreover, 237 out of the 293 LAC specimens (80.9%) showed high POLQ protein expression levels (H-score: 150–300). We then investigated whether the difference in the POLQ protein expression level was associated with any clinicopathological factors in the LAC patients. The results showed high POLQ protein expression levels were associated with a positive lymph node status and higher TNM stages (Table 1). We also investigated whether the difference in the POLQ mRNA expression level was associated with any driver gene mutations in LAC using the TCGA database. The results showed that the POLQ mRNA expression level was associated with the *EGFR* mutation status (*p* = 0.0047), but not with the *KRAS* or *BRAF* mutation status; POLQ overexpression was more frequently found in *EGFR* wild-type (WT) tumors than in *EGFR* mutation-positive tumors (81.1% vs. 50.0%) (Table 2). These results suggest that POLQ is overexpressed in a large subset of LAC cases and that POLQ overexpression in LAC is associated with advanced pathologic stage, lymph node metastasis, and *EGFR*-WT status. 

### 2.2. Association of Increased POLQ Expression with Increased Somatic Mutation Load in LAC

**Figure 2:**
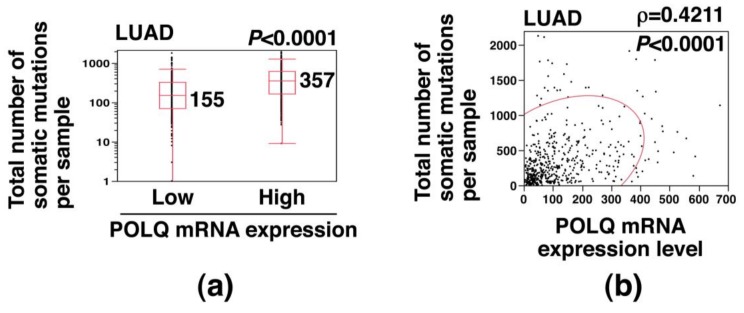
Association of increased POLQ expression with the somatic mutation load in LAC, determined using the data (*n* = 513) from the TCGA database (ID: LUAD). (**a**) Comparison of the total number of somatic mutations between a group of cancers showing high POLQ expression levels and another group showing low POLQ expression levels among cases of LAC. A box-plot analysis showed a statistically significant difference in the number of somatic mutations between the two groups (*p* < 0.0001, Mann–Whitney *U* test). The median values are shown. (**b**) Scatterplot showing a positive correlation between the POLQ mRNA expression level and the total number of somatic mutations in LAC. The Spearman rank correlation coefficient (ρ) and *p*-value are shown; a bivariate normal ellipse (*p* = 0.95) was obtained.

We next investigated the effect of POLQ overexpression on the somatic mutation burden in LAC, using data from the TCGA database. The total number of somatic mutations was significantly higher in the high POLQ expression group than in the low POLQ expression group (median: 357 vs. 155; *p* < 0.0001) (Figure 2a). Moreover, the total number of somatic mutations showed a statistically significant positive correlation with the POLQ mRNA expression level (ρ = 0.4211; *p* < 0.0001) (Figure 2b). These results suggest that increased POLQ expression is associated with an increased somatic mutation load in LAC.

### 2.3. Comparison of the Sensitivity to DNA-Damaging Agent and Frequency of Mutations among Lung Cancer Cells Showing Different Expression Levels of POLQ

**Figure 3:**
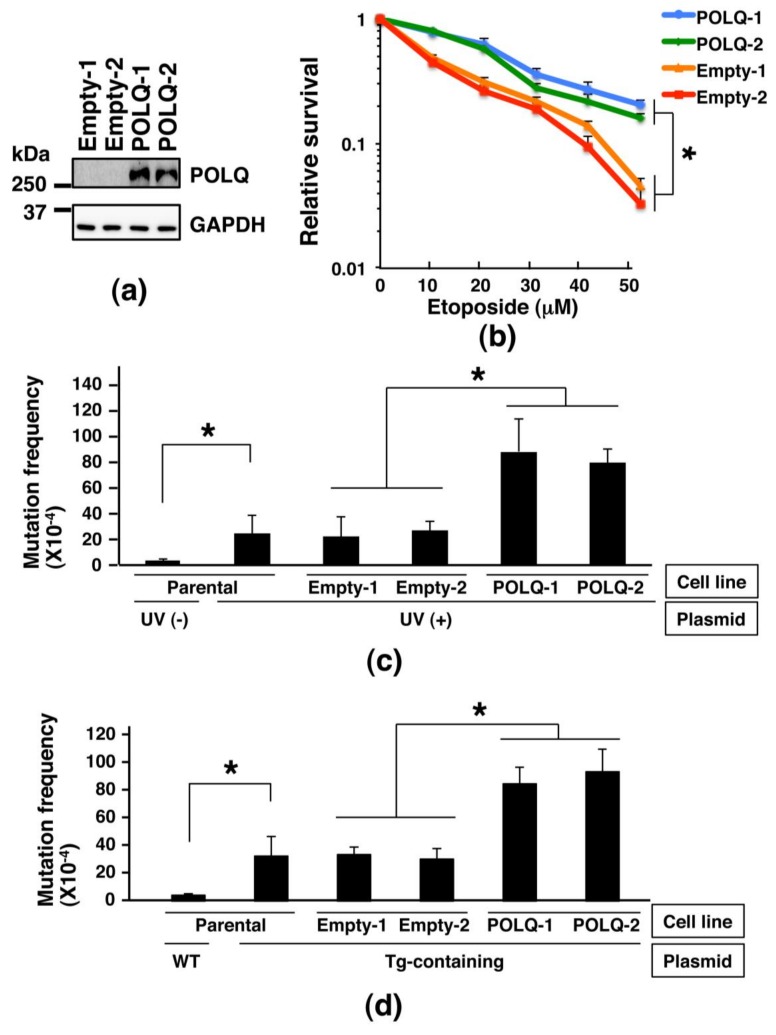
Comparison of the sensitivities to DNA-damaging agent and mutation frequency between lung cancer cells showing different POLQ expression levels. (**a**) Detection of FLAG-POLQ proteins in cumate-inducible stable H1299 lung cancer cell lines (POLQ-1 and -2) designed to express FLAG-POLQ in the presence of cumate; the POLQ proteins were detected by Western blot analysis. Empty vector-transposed cells (Empty-1 and -2) were used as control. GAPDH expression was also analyzed as the internal control. (**b**) Clonogenic survival assay following treatment with etoposide. The survival fraction was compared between the empty vector-transposed H1299 clones and H1299 clones showing inducible POLQ expression. Data are means + standard deviation (SD) of three independent experiments. (**c**) Measurement of the mutation frequency of the *supF* gene in the pMY189 shuttle plasmid, using a *supF* forward mutation assay with pMY189 treated or not treated with UV light in the parental H1299 or H1299-derived stable clones. Data are means + SD of >3 independent experiments. (**d**) Measurement of the mutation frequency of the *supF* gene in pMY189, using a *supF* forward mutation assay with Tg-containing pMY189 in parental H1299 or H1299-derived stable clones. Data shown are means + SD of ≥3 independent experiments. Statistical analyses were performed using an unpaired *t*-test and the asterisks (*) show a statistically significant difference (**b–d**).

We next planned to investigate the effects of POLQ overexpression in human lung cancer cells. First, we established H1299 lung cancer cell lines capable of inducibly expressing the POLQ protein and control H1299 cell lines using the PiggyBac transposon vector system (Figure 3a). Then, we compared the sensitivity of empty vector-transposed clones and POLQ-transposed clones to the DNA DSB-inducing chemical etoposide. The results showed that POLQ-transposed clones were more resistant to etoposide than the empty vector-transposed clones (Figure 3b). When the average surviving fractions of the two types of clones after exposure to 50 μM etoposide were compared, the surviving fraction of the POLQ-overexpressing clones was 4.6-fold greater than that of the empty vector-transposed clones (*p* < 0.01 for all). These results suggest that lung cancer cells with higher POLQ expression levels are more resistant to DSBs than lung cancer cells with lower POLQ expression levels.

Since analysis of data from the TCGA dataset revealed an association of increased POLQ expression with an increased somatic mutation load in LAC (Figure 2), we investigated whether POLQ overexpression actually induced mutations in the lung cancer cell clones established by us. Towards this objective, we planned a *supF* forward mutation assay in a situation that mutation frequency is suspected to be increased by the use of a ultraviolet (UV)-irradiated or thymine glycol (Tg)-containing shuttle plasmid, since it has been reported that POLQ is involved in the TLS of Tg and DNA base damage induced by UV [12,26]. First, a *supF* forward mutation assay using UV-irradiated pMY189 was performed in the parental H1299 cells, which showed that the mutation frequency was significantly increased by UV irradiation (average: 3.0 × 10^−4^ vs. 2.5 × 10^−3^; *p* < 0.01) (Figure 3c). Comparison of the mutation frequency determined by the *supF* forward mutation assay using UV-irradiated pMY189 between the empty vector-transposed clones and POLQ-transposed clones revealed a significantly higher mutation frequency in the POLQ-transposed clones than in the empty vector-transposed clones (average of two clones: 8.4 × 10^−3^ vs. 2.5 × 10^−3^; *p* < 0.01 for all) (Figure 3c). Then, a *supF* forward mutation assay using Tg-containing pMY189 was performed in the parental H1299 cells; the mutation frequency was significantly increased by the introduction of Tg (average: 3.2 × 10^−3^ vs. 4.1 × 10^−4^; *p* < 0.001) (Figure 3d). When the mutation frequency using the Tg-containing shuttle plasmid was compared between the empty vector-transposed clones and POLQ-transposed clones, the frequency was significantly higher in the POLQ-transposed clones than in the empty vector-transposed clones (average of two clones: 8.9 × 10^−3^ vs. 3.2 × 10^−3^; *p* < 0.01 for all) (Figure 3d). These results suggest that POLQ overexpression is associated with an increase in the frequency of mutations induced by UV irradiation and Tg introduction in lung cancer cells.

### 2.4. Concurrent POLQ and PLK4 Overexpression in LAC and Induction of Centrosome Amplification

**Figure 4:**
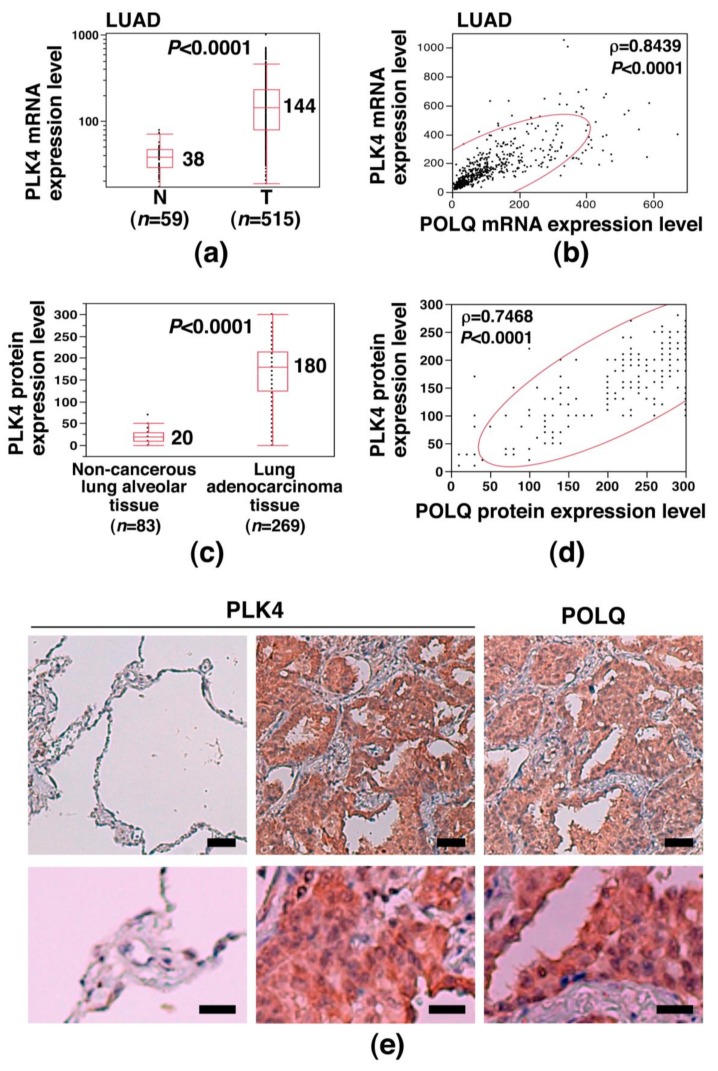
Concurrent overexpression of POLQ and PLK4 in primary LAC. (**a**) PLK4 mRNA overexpression detected in LAC using data from the TCGA database (ID: LUAD). Statistical comparison was performed using a Mann–Whitney *U* test between non-cancerous tissue (N) and cancerous tissue (T); median expression levels and the *p*-value are shown. (**b**) A significant positive correlation between the POLQ and PLK4 mRNA expression levels in LAC. The Spearman rank correlation coefficient (ρ) and *p*-value are provided. A bivariate normal ellipse (*p* = 0.95) was observed. (**c**) Overexpression of PLK4 protein in LAC, determined by IHC analysis using rabbit anti-PLK4 polyclonal antibody in cases of our hospital (HUH). A Mann–Whitney *U* test was used for statistical comparison between non-cancerous lung alveolar tissue (N) and LAC tissue (T); the *p*-value and median expression levels are shown. (**d**) A significant positive correlation was detected between the POLQ and PLK4 protein expression levels in LAC. The data on the POLQ protein expression level were derived from the data shown in Figure 1b,c. The Spearman rank correlation coefficient (ρ) and *p*-value are shown. A bivariate normal ellipse (*p* = 0.95) was obtained. (**e**) Representative IHC results of co-overexpression of PLK4 and POLQ proteins in a case of LAC. The leftmost panel represents the results in non-cancerous lung tissue, while the remaining show the results in LAC from the same patient. The lower panels show a part of the upper panels at a higher magnification. Scale bar = 50 μm (upper); 20 μm (lower). Another set of representative results is shown in Supplementary Figure S1.

To further investigate the characteristics of POLQ-overexpressing lung cancer cells, the mRNA expression data of the LAC cases from the TCGA database were carefully examined. We found that the mRNA expression of PLK4, a gene that is involved in the centrosome regulation [27,28], was significantly upregulated in LAC (median: 144 vs. 38; *p* < 0.0001) (Figure 4a), and that the expression levels of POLQ mRNA were significantly positively correlated with those of PLK4 mRNA (ρ = 0.8439, *p* < 0.0001) (Figure 4b). To examine if PLK4 and POLQ were also overexpressed at the protein level, we performed an IHC analysis using anti-PLK4 antibody in the primary LAC cases used for the experiment shown in Figure 1b,c. The results revealed significantly higher expression levels of PLK4 protein in the cancerous tissues than in the non-cancerous lung alveolar tissues (median: 180 vs. 20; *p* < 0.0001) (Figure 4c) and also that the expression levels of POLQ and PLK4 proteins were significantly positively correlated (ρ = 0.7468, *p* < 0.0001) (Figure 4d; a representative IHC result is shown in Figure 4e and Supplementary Figure S1). These results suggest that POLQ and PLK4 are concurrently overexpressed in LAC.

**Figure 5:**
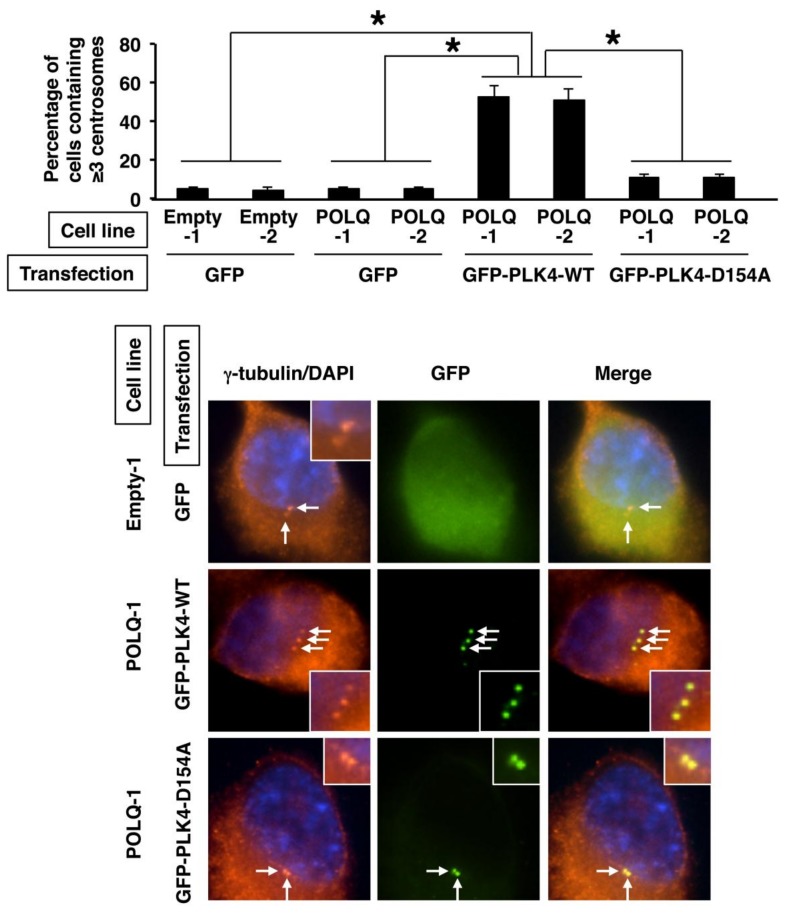
Centrosome amplification in the lung cancer cells with concurrent overexpression of POLQ and PLK4. The H1299 lung cancer cell line-derived cells (Empty-1, Empty-2, POLQ-1, and POLQ-2) were transiently transfected with plasmid for expression of GFP or GFP-PLK4 (green) and 48 h post-transfection, the cells were immunostained with anti-γ-tubulin antibody (red) and the nuclei were stained with DAPI (blue). The percentage of cells with ≥3 centrosomes was measured among the GFP-positive (or GFP-PLK4-positive) cells, and is shown in the upper bar graph. Data shown are as the means and standard errors derived from three independent experiments. Statistical analyses were performed using an unpaired *t*-test and the asterisks (*) denote a statistically significant difference. Representative immunostained images are shown in the lower panels. The arrows show the positions of the centrosomes, and the insets show magnified images of the areas indicated by the arrows.

Since PLK4 is known to be involved in centrosome regulation [27,28], we then investigated whether the number of centrosomes was dysregulated in the lung cancer cells showing concurrent overexpression of POLQ and PLK4. Especially, as centrosome amplification is known as one of the malignant phenotypes in subsets of cancers, the percentage of cells showing centrosome amplification was examined by immunofluorescence analysis of γ-tubulin, a major centrosomal protein [29]. The results showed that the percentage of cells showing centrosome amplification among the GFP-positive or GFP-PLK4-positive cells was significantly higher in the POLQ-transposed cells transiently transfected with the GFP-PLK4-WT expression vector (i.e., both POLQ- and GFP-PLK4-WT-overexpressing cells) than in the empty vector-transposed cells or POLQ-overexpressing cells transiently transfected with only the GFP expression vector (*p* < 0.01 for all) (Figure 5). Moreover, when catalytically inactive (kinase-dead) mutant PLK4-D154A was utilized, the percentage of cells showing centrosome amplification was significantly lower in both the POLQ- and GFP-PLK4-D154A-overexpressing cells than in the POLQ- and GFP-PLK4-WT-overexpressing cells (*p* < 0.01 for all) (Figure 5). These results suggest that centrosome amplification is induced in lung cancer cells showing concurrent overexpression of POLQ and PLK4.

### 2.5. Association of Increased POLQ Expression with an Increased Somatic Mutation Load and PLK4 Overexpression in Diverse Human Cancers

**Figure 6:**
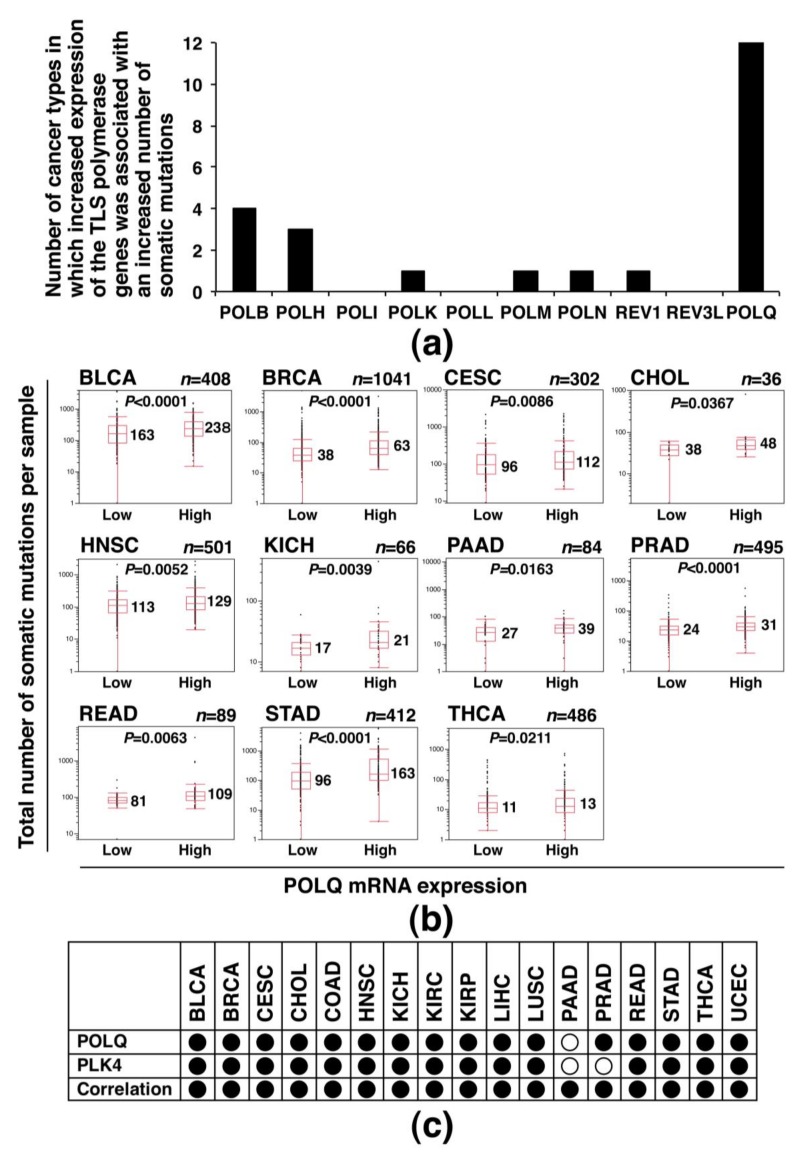
Association of POLQ overexpression with the somatic mutation load and PLK4 overexpression in diverse human cancers, determined using data from the TCGA database. (**a**) Comparison of the number of cancer types showing increased expression of the TLS polymerase gene associated with an increased number of somatic mutations. Ten TLS polymerase genes were examined for the number in 18 cancer types, with Mann–Whitney *U* test used to compare the total number of somatic mutations between a group of cancers showing low TLS polymerase expression and a group of cancers showing high TLS polymerase gene expression. (**b**) Results of box-plot analyses of cancer types showing increased expression of the POLQ gene associated with an increased number of somatic mutations in (**a**). Among the 12 cancer types which showed a significant association, the results for LAC is already shown in Figure 2, and the results for the remaining 11 cancer types are shown. The median mutation number in each group and the *p*-values are shown in the graph. Results of the box-plot analyses of cancer types showing increased expression of the TLS polymerase gene other than POLQ associated with an increased number of somatic mutations in (**a**) are shown in Supplementary Figure S2. (**c**) Concurrent overexpression of POLQ and PLK4 in diverse human cancers. Cancer types in which statistical significance was observed in relation to POLQ overexpression and PLK4 overexpression, and a positive correlation was observed between the expression levels of POLQ and PLK4, which are marked with filled circles; other cancer types are marked with clear circles. Detailed data are shown in Supplementary Figure S3.

Finally, to investigate whether the association between increased POLQ expression and increased somatic mutation load is also common in other human cancers and, if so, whether such an association is specific among various TLS polymerase genes [30], we used the TCGA dataset to examine the mRNA expression levels of 10 TLS polymerase genes (POLB, POLH, POLI, POLK, POLL, POLM, POLN, REV1, REV3L, and POLQ; Supplementary Table S1) and total number of somatic mutations across a panel of 18 distinct cancer types, including LAC. For this examination, the total numbers of somatic mutations were compared between the cancers showing low TLS polymerase gene expression and those showing high TLS polymerase gene expression using the Mann–Whitney *U* test (case number of each cancer type is summarized in Supplementary Table S2). The results showed that the number of cancer types in which increased expression of the TLS polymerase genes was associated with an increased number of somatic mutations was the highest (the number = 12) for the *POLQ* gene among the 10 TLS polymerase genes (Figure 6a; the box plot analyses of cancer types in which a significant association was observed are shown in Figure 6b). On the other hand, the number of cancer types showing such an association was only 0–4 for the other TLS polymerase genes (Figure 6a, Supplementary Figure S2). The above results suggest an association between overexpression and an increased somatic mutation load in diverse human cancers for only the *POLQ* gene among the TLS polymerase genes.

Additionally, we examined whether concurrent overexpression of POLQ and PLK4, which was detected in LAC (Figure 4), is also observed in various other human cancers using the data of 17 cancer types from the TCGA database. The results revealed POLQ overexpression (significant difference: 16/17 (94.1%)), PLK4 overexpression (significant difference: 15/17 (88.2%)), and a significant positive correlation between POLQ and PLK4 expression (17/17 (100%)) in the cancer types (Figure 6c, Supplementary Figure S3), suggesting that POLQ and PLK4 are concurrently overexpressed in diverse types of human cancers.

## 3. Discussion

In the present study, we found overexpression of both POLQ mRNA and POLQ protein in LAC, and that increased POLQ expression was associated with an advanced pathologic stage, an *EGFR*-WT status, and an increased somatic mutation load in LAC. When H1299 lung cancer cell clones overexpressing POLQ were established and examined for their malignant potential, the clones showed greater resistance to a DSB-inducing chemical and an increased number of mutations. Further analysis revealed that POLQ overexpression was positively correlated with PLK4 overexpression in LAC and that PLK4 overexpression in the POLQ-overexpressing H1299 was associated with the induction of centrosome amplification. Interestingly, POLQ overexpression was associated with an increased somatic mutation load and PLK4 overexpression in diverse human cancers, while overexpressions of the nine TLS polymerases other than POLQ examined were associated with an increased somatic mutation load at much lower frequencies. These results suggest that POLQ overexpression is associated with advanced pathologic stage, increased somatic mutation load, and PLK4 overexpression, the latter inducing centrosome amplification, in LAC. This is the first paper to identify the pathobiological characteristics of LAC showing POLQ overexpression, providing a significant and important link between abnormal expressions of error-prone DNA polymerases and LAC.

In this study, POLQ overexpression was demonstrated at both the mRNA level and protein level in LAC. Although the proportion of LACs showing elevated POLQ mRNA expression levels differed between previous two reports [20,24], our finding of overexpression of POLQ mRNA in the majority of the examined cases is consistent with the results reported by Allera-Moreau et al. [24]. In regard to the POLQ protein expression, our IHC analysis revealed, for the first time, overexpression of POLQ protein in LAC. Moreover, POLQ protein overexpression was also detected at a high frequency in the examined cases. Thus, POLQ overexpression is considered as a common event in LAC.

The results of the TCGA-based analysis revealed the existence of a strong association between POLQ overexpression and an increased somatic mutation load in LAC. In addition, lung cancer cells with higher POLQ expression levels showed a higher frequency of mutations induced by UV irradiation and Tg introduction. These findings are being demonstrated for the first time and are compatible with the previously identified fact that the POLQ DNA polymerase is involved in error-prone TLS and error-prone TMEJ [9,10,11,12,13,14,15,16,17,18]. Although at present it remains unknown which biological events, i.e., TMEJ or TLS in situations other than TMEJ, contribute more profoundly to mutation induction, our findings strongly suggest that overexpression of POLQ, but not of other TLS polymerases, is a potent factor in inducing mutations in LAC. Consistent with this notion, association of the POLQ expression level with the number of non-synonymous mutations has also been reported in carcinomas of the breast, ovary, and uterus [17]. Thus, like inactivation of mismatch repair genes (MLH1 and MSH2), BRCA genes (BRCA1 and BRCA2), and DNA polymerase genes (POLE), overexpression of APOBEC3B, and reduced expression of DNA glycosylase genes (NEIL1, NEIL2, and MUTYH) [31,32,33,34,35], POLQ overexpression is likely to be another cancer-related gene abnormality associated with mutagenesis of the entire coding genome. Since somatic mutations in cancer-associated genes can lead to an exacerbation of the malignant potential, POLQ overexpression could be a key event in the pathogenesis of LAC. From another point of view, since it has recently been revealed that tumor mutation load is one of the factors determining the response to immune checkpoint inhibitors (ICIs) [36], the POLQ expression level could determine the response to ICIs via defying the number of tumor somatic mutations, and ICIs could be more effective against LACs exhibiting POLQ overexpression. Future investigations are needed to clarify this possibility.

The present study is the first to show concurrent overexpression of POLQ and PLK4 at both the mRNA and protein level in LAC, and also induction of centrosome amplification in lung cancer cells associated with POLQ and PLK4 overexpression. Our finding of PLK4 mRNA overexpression in LAC is consistent with the results of the very recent work conducted by Kawakami et al. [37]. Centrosome amplification is a common feature of LAC [38,39], and centrosome amplification is associated with an increased frequency of merotelic kinetochore-microtubule attachment errors, lagging chromosome formation, and aberrant mitotic spindle formation, all of which can cause chromosomal instability [40,41,42]. In addition, centrosome amplification is also known to be involved in cellular invasion [43]. Since PLK4 controls centrosome duplication in human cells, it is considered that PLK4 overexpression caused the centrosome amplification in the lung cancer cells of our system, consistent with the observations in previous studies in cells other than lung cancer cells [27,44,45,46]. Thus, it is suspected that centrosome amplification in LAC could be attributable, at least in part, to PLK4 overexpression and that the malignant phenotypes resulting from centrosome amplification mentioned above could be involved in the pathogenesis of LAC.

The findings of our present study revealed an association between POLQ protein overexpression and advanced pathologic stage/lymph node metastasis in LAC. Since our study also showed an association of POLQ overexpression in lung cancer cells with mutation induction, PLK4 overexpression, which contributes to centrosome amplification, and resistance to DSBs, it is speculated that POLQ overexpression contributes to increasing the malignant potential of LAC. Interestingly, association of POLQ overexpression with increased somatic mutation load and PLK4 overexpression has been observed in other many cancer types, suggesting that POLQ overexpression may be involved in the pathogenesis of diverse human cancers. In addition, we would like to point out that the association of overexpression with an increased number of somatic mutations was observed at a low frequency for the 9 TLS polymerases examined, other than POLQ, in the 18 cancer types included in the analysis. This finding suggests that while these polymerases are known to mediate error-prone TLS in some situations, which may differ among the TLS polymerases [30], the notion that cancer cells overexpressing TLS polymerases harbor an increased number of somatic mutations may not always be accurate. It is likely that POLQ is a unique TLS polymerase gene from the standpoint of the somatic mutation burden in carcinomas.

In our present study, we investigated the relationship between the POLQ mRNA expression levels and the status of driver gene mutations in LAC, which revealed that POLQ overexpression was associated with the *EGFR*-WT status. While treatment with EGFR tyrosine kinase inhibitors (TKIs) has been reported as effective strategy in patients with *EGFR*-mutant non-small cell lung carcinoma, including LAC [47], it would seem that LAC patients with tumor POLQ overexpression, which was found to be associated with *EGFR*-WT status, are not likely to benefit from EGFR-TKI treatment, as compared to LAC patients without POLQ overexpression in the tumor. 

The limitations of this research are as follows: (1) Usage of only one lung cancer cell line showing different expression levels of POLQ. We established H1299 lung cancer cell lines capable of inducibly expressing the POLQ protein and control H1299 cell lines using the PiggyBac transposon vector system, and performed three kinds of experiments using these cell lines. We obtained significant differences in the results between the POLQ-overexpressing clones and empty-vector-transposed clones. However, use of multiple kinds of lung cancer cell lines may have provided more definitive evidence. Especially, the H1299 cells harbor a mutation of the *TP53* tumor suppressor gene and are known as *TP53*-deficient cells [48]. Further experiments using a *TP53*-proficient lung cancer cell line would allow clarification of the roll of POLQ, irrespective of the *TP53* mutation status. In the future, we propose to extend the work to alleviate this shortcoming. (2) Lack of investigation of the relationship of POLQ expression with TTF-1 expression. TTF-1 is a transcription factor involved in surfactant production, morphogenesis, and differentiation in normal lung epithelial cells [49]; among LACs, it is mainly expressed in terminal respiratory unit (TRU)-type LAC [50]. Moreover, TTF-1 has recently been reported as a prognostic marker in patients with LAC [51]. Thus, it would be of interest to investigate the protein expression status of TTF-1 and its relationship with the POLQ protein expression level in LAC. In the future, we would like to investigate the relationship between the expression levels of POLQ and TTF-1.

## 4. Materials and Methods

### 4.1. Collection of Publicly Available Data

The mRNA expression and somatic mutation data for LAC [TCGA ID: LUAD] and 17 other cancer types were collected from the TCGA data portal. All the cancer types included in this study are listed in Supplementary Table S2. The expression data were obtained in the form of RNA-seq by Expectation Maximization (RSEM). An expression level of more than three-fold as compared to the median value in non-cancerous lung tissue specimens was defined as overexpression. The somatic mutation data by whole-exome sequencing were obtained and are shown in the form of a mutation annotation format file. MuTect was utilized for the variant-calling step. In regard to the status of driver gene (*EGFR*, *KRAS*, and *BRAF*) mutations in LAC, TCGA data were downloaded from the cBioPortal [52] (*n* = 230). We defined cases harboring *EGFR* exon 19 deletions or L858R mutation as *EGFR* mutation-positive case [53]. We also defined cases harboring *KRAS* codon 12, 13, or 61 mutations and *BRAF* V600E mutation as *KRAS* mutation-positive and *BRAF* mutation-positive cases, respectively [54,55]. When the association between increased POLQ expression and increased somatic mutation load was investigated in 18 cancer types, the median expression value in the cancer tissue samples was used as the cut-off value to dichotomize the cancer cases according to the expression levels of the TLS polymerase genes. 

### 4.2. Primary LAC Specimens

IHC analysis was performed using paraffin-embedded blocks of LAC tissues obtained from a total of 293 patients with primary LAC at our hospital (Hamamatsu University Hospital (HUH)). The present study design was approved by the Institutional Review Board of the Hamamatsu University School of Medicine (15-067) and the present study was carried out in accordance with the World Medical Association Declaration of Helsinki.

### 4.3. IHC Analysis

Paraffin sections were incubated with a rabbit anti-POLQ polyclonal antibody (EnoGene, New York, NY, USA) or a rabbit anti-PLK4 polyclonal antibody (Proteintech, Chicago, IL, USA), followed by incubation with Histofine Simple Stain MAX PO (Nichirei, Tokyo, Japan). The visualization of antigen-antibody complex was performed using 3,3′-diaminobenzidine tetrahydrochloride. To assess the protein expression level, the modified H-scores were calculated by multiplying the intensity value (0, absent; 1, weak; 2, moderate; 3, strong: representative images are shown in Supplementary Figure S4) in the cytoplasm by the percentage of cells with each intensity value (0–100%), to obtain values of 0–300. The H-score of 150 was used to as the cutoff value to dichotomize the cancer cases based on the H-score.

### 4.4. Cell Cultures

The human lung cancer cell line H1299 was purchased from the American Type Culture Collection (ATCC; Manassas, VA, USA). H1299 cells and their stable inducible cell clones were maintained at 37 °C in RPMI1640 medium (Sigma-Aldrich, St. Louis, MO, USA) supplemented with 10% fetal bovine serum (Sigma-Aldrich) and penicillin/streptomycin (Gibco Laboratories, Grand Island, NY, USA), under a 5% CO_2_ atmosphere.

### 4.5. Establishment of Stable Inducible Cell Lines

The FLAG-POLQ was amplified using a polymerase chain reaction with PrimeSTAR HS DNA polymerase (Takara, Kyoto, Japan) and the POLQ expression vector [21], kindly provided by Dr. J.S. Hoffmann (Le Centre de Recherches en Cancérologie de Toulouse, Université Toulouse, France), as a template; the amplified sequence was then inserted into a PiggyBac cumate switch inducible vector (System Biosciences, Mountain View, CA, USA) at the *Not*I restriction enzyme site. H1299 cells were transfected with the POLQ expression construct together with the PiggyBac transposase vector (System Biosciences); positively transposed cells were then selected using puromycin (1.6 μg/mL: Clontech, Palo Alto, CA, USA). We also prepared cells transfected with an empty (parental) PiggyBac cumate switch inducible vector and the transposase vector. In total, two POLQ-transposed clones (named POLQ-1 and -2) and two empty vector-transposed clones (named Empty-1 and -2) were established.

### 4.6. Western Blot Analysis

Cultured cells were lysed in a buffer containing 50 mM HEPES-KOH (pH 7.5), 150 mM NaCl, 0.1% sodium dodecyl sulfate, 1% Triton X-100, 50 mM sodium fluoride, 1 mM sodium orthovanadate, 0.5% sodium deoxycholate, and protease inhibitor cocktail (Sigma-Aldrich). A Western blot analysis was then performed using a rabbit anti-FLAG polyclonal antibody (Sigma-Aldrich), or a mouse anti-GAPDH monoclonal antibody (clone 6C5; Abcam, Cambridge, UK). Immunoreactivity was visualized using a ChemiDoc Touch imaging system (Bio-Rad, Hercules, CA, USA) and an ECL chemiluminescence system (GE Healthcare Bio-Science, Piscataway, NJ, USA). Whole blots, molecular markers, and densitometric intensity ratios are shown in Supplementary Figure S5.

### 4.7. Clonogenic Survival Assay

Etoposide (Sigma-Aldrich) was diluted to experimental concentrations from a 50 mM stock in dimethyl sulfoxide (DMSO). Treatment with etoposide (0–50 μM) was carried out at 20 h after cell plating. After 1-h exposure to the drug, cells were rinsed thrice with PBS, followed by the addition of regular medium. After 13 days, methanol fixation and staining with methylene blue were performed to identify visible colonies (≥50 cells). The surviving fractions were calculated as the plating efficiency of the treated cells relative to that of the untreated control cells.

### 4.8. Preparation of Shuttle Vector Plasmids

The pMY189 is a shuttle vector plasmid containing the bacterial suppressor tRNA (*supF*) gene [56] and it was used for a *supF* forward mutation assay. Before the assay, two kinds of damaged base(s)-containing pMY189 (i.e., UV-irradiated pMY189 and Tg-containing pMY189) were prepared. For UV-irradiated pMY189, purified plasmid DNA was exposed to UV (700 J/m^2^) using a UV crosslinker (UVP, Upland, CA, USA). For Tg-containing pMY189, first, single-stranded pMY189 DNA was prepared using *E. coli* XL1-Blue MRF′ (Stratagene, La Jolla, CA, USA) and R408 Helper Phage (Stratagene), and in a reaction mixture 30 μg of the single-stranded plasmid pMY189 and a five-fold molar excess of 5′-phosphorylated 25-mer oligonucleotide containing a single *5R,6S*-Tg at nucleotide position 134 of the *supF* gene [5′-GGA GCA GAC TC(Tg) AAA TCT GCC GTC A-3′] (Japan Bio Services, Saitama, Japan) were annealed. Forty units of T4 DNA polymerase (Takara), 2400 units of T4 DNA ligase (New England Biolabs, Beverly, MA, USA), 1 mM of ATP, and 600 μM of deoxynucleotide triphosphate were added to the reaction mixture and the mixture was incubated at 37 °C for 4 h. Then, closed circular double-stranded pMY189 containing a Tg was isolated using cesium chloride-ethidium bromide density gradient centrifugation. 

### 4.9. SupF Forward Mutation Assay

Cells were transfected with pMY189 plasmids containing or not containing damaged base(s) using the LipofectAMINE 2000 reagent (Invitrogen, Carlsbad, CA, USA). The subsequent steps were performed as described previously [57,58,59]. The mutation frequencies were calculated as the number of *E. coli supF* mutants per total number of *E. coli* transformants.

### 4.10. Indirect Immunofluorescence Analysis

Cells were transfected with GFP-PLK4 (WT and D154A-mutant) [27] expression vector, which were received as a kind gift from Dr. E.A. Nigg (Max-Planck-Institute for Biochemistry, Germany; University of Basel, Switzerland), or the GFP-alone expression vector. After 48 hours, the cells were incubated with anti-γ-tubulin antibody (GTU88; Sigma-Aldrich). AlexaFluor 594-conjugated goat anti-mouse IgG (Molecular Probes, Eugene, OR, USA) was used as secondary antibody, and 4′,6-diamidino-2-phenylindole (DAPI) was used to counterstain DNA. Centrosome amplification was defied as ≥3 centrosomes (γ-tubulin-positive signal) per cell.

### 4.11. Statistical Analysis

The statistical analyses were performed using a Mann–Whitney *U* test, a Spearman rank correlation test, an unpaired *t*-test, and a Fisher’s exact test. JMP version 9.0 software (SAS Institute, Cary, NC, USA) or GraphPad QuickCalcs (GraphPad Software Inc., San Diego, CA, USA) was used for the statistical analyses. *p*-values of less than 0.05 were considered as denoting statistical significance.

## 5. Conclusions

Our present findings suggest that POLQ overexpression is associated with advanced pathologic stage, increased somatic mutation load, and PLK4 overexpression, the latter causing centrosome amplification, in LAC.

## Figures and Tables

**Table 1:** Association between the POLQ protein expression level and clinicopathological factors in 293 patients with primary lung adenocarcinoma.

Factor	No. of Cases	POLQ Protein Expression Level ^§^		*p*-Value ^†^
		Low (*n* = 56)	High (*n* = 237)	
Age (y)				
<60	82	19 (23.2%)	63 (76.8%)	0.3206
≥60	211	37 (17.5%)	174 (82.5%)	
Sex				
Female	123	23 (18.7%)	100 (81.3%)	1.0000
Male	170	33 (19.4%)	137 (80.6%)	
Smoking				
Non-smoker	99	21 (21.2%)	78 (78.8%)	0.1510
Smoker	128	17 (13.3%)	111 (86.7%)	
pT				
pT1/pT2	258	51 (19.8%)	207 (80.2%)	0.6463
pT3/pT4	35	5 (14.3%)	30 (85.7%)	
pN				
pN0	218	50 (22.9%)	168 (77.1%)	0.0012
pN1-pN3	69	4 (5.8%)	65 (94.2%)	
TNM Stage				
I/II	240	54 (22.5%)	186 (77.5%)	0.0008
III/IV	53	2 (3.8%)	51 (96.2%)	

^§^ Low, H-score <150; High, H-score ≥150; ^†^ Fisher’s exact test.

**Table 2:** Relationship between POLQ mRNA expression and the mutation status of driver genes in 230 cases of primary lung adenocarcinoma using data from the TCGA database.

Factor	No. of Cases	POLQ mRNA Expression Level		*p*-Value ^†^
		Low (*n* = 49)	High (*n* = 181)	
*EGFR*				
WT	212	40 (18.9%)	172 (81.1%)	0.0047
Mutation	18	9 (50.0%)	9 (50.0%)	
*KRAS*				
WT	156	31 (19.9%)	125 (80.1%)	0.4915
Mutation	74	18 (24.3%)	56 (75.7%)	
*BRAF*				
WT	225	48 (21.3%)	177 (78.7%)	1.0000
Mutation	5	1 (20.0%)	4 (80.0%)	

^†^ Fisher’s exact test.

## Supplementary Materials

The following are available online at https://www.mdpi.com/2072-6694/11/5/722/s1, Table S1. List of translesion DNA synthesis (TLS) polymerase genes analyzed in this study; Table S2. Tumor types from the TCGA database analyzed in this study; Figure S1. Another representative set of results of IHC analysis for concurrent overexpression of PLK4 and POLQ proteins in a case of LAC; Figure S2. Results of the box-plot analyses of cancer types showing increased expression of the TLS polymerase gene other than POLQ associated with an increased number of somatic mutations using data from the TCGA database; the data is linked with Figure 6a; Figure S3. Detection of concurrent overexpression of POLQ and PLK4 in diverse human cancers determined using data from the TCGA database; the data is linked with Figure 6c; Figure S4. Representative images of the staining intensities in the IHC analysis; Figure S5. Blots, molecular markers, and densitometric intensity ratios in Western blot analysis.

## References

[1] Bray F., Ferlay J., Soerjomataram I., Siegel R.L., Torre L.A., Jemal A. (2018). Global cancer statistics 2018: GLOBOCAN estimates of incidence and mortality worldwide for 36 cancers in 185 countries. CA Cancer J. Clin..

[2] Galateau-Salle F., Churg A., Roggli V., Travis W.D. (2016). The 2015 World Health Organization Classification of Tumors of the Pleura: Advances since the 2004 Classification. J. Thorac. Oncol..

[3] Inamura K. (2018). Update on immunohistochemistry for the diagnosis of lung cancer. Cancers.

[4] Cancer Genome Atlas Research Network (2014). Comprehensive molecular profiling of lung adenocarcinoma. Nature.

[5] Alexandrov L.B., Ju Y.S., Haase K., Van Loo P., Martincorena I., Nik-Zainal S., Totoki Y., Fujimoto A., Nakagawa H., Shibata T. (2016). Mutational signatures associated with tobacco smoking in human cancer. Science.

[6] Shi J., Hua X., Zhu B., Ravichandran S., Wang M., Nguyen C., Brodie S.A., Palleschi A., Alloisio M., Pariscenti G. (2016). Somatic genomics and clinical features of lung adenocarcinoma: A retrospective study. PLoS Med..

[7] Wang Z., Wei Y., Zhang R., Su L., Gogarten S.M., Liu G., Brennan P., Field J.K., McKay J.D., Lissowska J. (2018). Multi-omics analysis reveals a HIF network and hub gene *EPAS1* associated with lung adenocarcinoma. EBioMedicine.

[8] Kundu S.T., Grzeskowiak C.L., Fradette J.J., Gibson L.A., Rodriguez L.B., Creighton C.J., Scott K.L., Gibbons D.L. (2018). TMEM106B drives lung cancer metastasis by inducing TFEB-dependent lysosome synthesis and secretion of cathepsins. Nat. Commun..

[9] Wood R.D., Doublié S. (2016). DNA polymerase 0 (POLQ), double-strand break repair, and cancer. DNA Repair (Amst.).

[10] Sallmyr A., Tomkinson A.E. (2018). Repair of DNA double-strand breaks by mammalian alternative end-joining pathways. J. Biol. Chem..

[11] Seki M., Marini F., Wood R.D. (2003). POLQ (Pol theta), a DNA polymerase and DNA-dependent ATPase in human cells. Nucleic Acids Res..

[12] Seki M., Masutani C., Yang L.W., Schuffert A., Iwai S., Bahar I., Wood R.D. (2004). High-efficiency bypass of DNA damage by human DNA polymerase Q. EMBO J..

[13] Arana M.E., Seki M., Wood R.D., Rogozin I.B., Kunkel T.A. (2008). Low-fidelity DNA synthesis by human DNA polymerase theta. Nucleic Acids Res..

[14] Roerink S.F., van Schendel R., Tijsterman M. (2014). Polymerase theta-mediated end joining of replication-associated DNA breaks in *C. elegans*. Genome Res..

[15] Yousefzadeh M.J., Wyatt D.W., Takata K., Mu Y., Hensley S.C., Tomida J., Bylund G.O., Doublié S., Johansson E., Ramsden D.A. (2014). Mechanism of suppression of chromosomal instability by DNA polymerase POLQ. PLoS Genet..

[16] Koole W., van Schendel R., Karambelas A.E., van Heteren J.T., Okihara K.L., Tijsterman M. (2014). A Polymerase Theta-dependent repair pathway suppresses extensive genomic instability at endogenous G4 DNA sites. Nat. Commun..

[17] Ceccaldi R., Liu J.C., Amunugama R., Hajdu I., Primack B., Petalcorin M.I., O’Connor K.W., Konstantinopoulos P.A., Elledge S.J., Boulton S.J. (2015). Homologous-recombination-deficient tumours are dependent on Polθ-mediated repair. Nature.

[18] Mateos-Gomez P.A., Gong F., Nair N., Miller K.M., Lazzerini-Denchi E., Sfeir A. (2015). Mammalian polymerase θ promotes alternative NHEJ and suppresses recombination. Nature.

[19] Ranjha L., Howard S.M., Cejka P. (2018). Main steps in DNA double-strand break repair: An introduction to homologous recombination and related processes. Chromosoma.

[20] Kawamura K., Bahar R., Seimiya M., Chiyo M., Wada A., Okada S., Hatano M., Tokuhisa T., Kimura H., Watanabe S. (2004). DNA polymerase theta is preferentially expressed in lymphoid tissues and upregulated in human cancers. Int. J. Cancer.

[21] Lemée F., Bergoglio V., Fernandez-Vidal A., Machado-Silva A., Pillaire M.J., Bieth A., Gentil C., Baker L., Martin A.L., Leduc C. (2010). DNA polymerase theta up-regulation is associated with poor survival in breast cancer, perturbs DNA replication, and promotes genetic instability. Proc. Natl. Acad. Sci. USA.

[22] Higgins G.S., Harris A.L., Prevo R., Helleday T., McKenna W.G., Buffa F.M. (2010). Overexpression of POLQ confers a poor prognosis in early breast cancer patients. Oncotarget.

[23] Pillaire M.J., Selves J., Gordien K., Gourraud P.A., Gentil C., Danjoux M., Do C., Negre V., Bieth A., Guimbaud R. (2010). A ‘DNA replication’ signature of progression and negative outcome in colorectal cancer. Oncogene.

[24] Allera-Moreau C., Rouquette I., Lepage B., Oumouhou N., Walschaerts M., Leconte E., Schilling V., Gordien K., Brouchet L., Delisle M.B. (2012). DNA replication stress response involving PLK1, CDC6, POLQ, RAD51 and CLASPIN upregulation prognoses the outcome of early/mid-stage non-small cell lung cancer patients. Oncogenesis.

[25] Lessa R.C., Campos A.H., Freitas C.E., Silva F.R., Kowalski L.P., Carvalho A.L., Vettore A.L. (2013). Identification of upregulated genes in oral squamous cell carcinomas. Head Neck.

[26] Seki M., Wood R.D. (2008). DNA polymerase theta (POLQ) can extend from mismatches and from bases opposite a (6–4) photoproduct. DNA Repair (Amst.).

[27] Habedanck R., Stierhof Y.D., Wilkinson C.J., Nigg E.A. (2005). The Polo kinase Plk4 functions in centriole duplication. Nat. Cell Biol..

[28] Arquint C., Nigg E.A. (2016). The PLK4-STIL-SAS-6 module at the core of centriole duplication. Biochem. Soc. Trans..

[29] Joshi H.C. (1994). Microtubule organizing centers and gamma-tubulin. Curr. Opin. Cell Biol..

[30] Sale J.E. (2013). Translesion DNA synthesis and mutagenesis in eukaryotes. Cold Spring Harb. Perspect. Biol..

[31] Roberts S.A., Lawrence M.S., Klimczak L.J., Grimm S.A., Fargo D., Stojanov P., Kiezun A., Kryukov G.V., Carter S.L., Saksena G. (2013). An APOBEC cytidine deaminase mutagenesis pattern is widespread in human cancers. Nat. Genet..

[32] Burns M.B., Temiz N.A., Harris R.S. (2013). Evidence for APOBEC3B mutagenesis in multiple human cancers. Nat. Genet..

[33] Alexandrov L.B., Nik-Zainal S., Wedge D.C., Aparicio S.A., Behjati S., Biankin A.V., Bignell G.R., Bolli N., Borg A., Børresen-Dale A.L. (2013). Signatures of mutational processes in human cancer. Nature.

[34] Shinmura K., Kato H., Kawanishi Y., Igarashi H., Goto M., Tao H., Inoue Y., Nakamura S., Misawa K., Mineta H. (2016). Abnormal Expressions of DNA Glycosylase Genes *NEIL1*, *NEIL2*, and *NEIL3* Are associated with somatic mutation loads in human cancer. Oxid. Med. Cell. Longev..

[35] Shinmura K., Kato H., Kawanishi Y., Yoshimura K., Igarashi H., Goto M., Tao H., Inoue Y., Sugiyama T., Furuse H. (2017). Reduced expression of the DNA glycosylase gene MUTYH is associated with an increased number of somatic mutations via a reduction in the DNA repair capacity in prostate adenocarcinoma. Mol. Carcinog..

[36] Braun D.A., Burke K.P., Van Allen E.M. (2016). Genomic Approaches to Understanding Response and Resistance to Immunotherapy. Clin. Cancer Res..

[37] Kawakami M., Mustachio L.M., Zheng L., Chen Y., Rodriguez-Canales J., Mino B., Kurie J.M., Roszik J., Villalobos P.A., Thu K.L. (2018). Polo-like kinase 4 inhibition produces polyploidy and apoptotic death of lung cancers. Proc. Natl. Acad. Sci. USA.

[38] Koutsami M.K., Tsantoulis P.K., Kouloukoussa M., Apostolopoulou K., Pateras I.S., Spartinou Z., Drougou A., Evangelou K., Kittas C., Bartkova J. (2006). Centrosome abnormalities are frequently observed in non-small-cell lung cancer and are associated with aneuploidy and cyclin E overexpression. J. Pathol..

[39] Shinmura K., Iwaizumi M., Igarashi H., Nagura K., Yamada H., Suzuki M., Fukasawa K., Sugimura H. (2008). Induction of centrosome amplification and chromosome instability in *p53*-deficient lung cancer cells exposed to benzo[a]pyrene diol epoxide (B[a]PDE). J. Pathol..

[40] Ganem N.J., Godinho S.A., Pellman D. (2009). A mechanism linking extra centrosomes to chromosomal instability. Nature.

[41] Cosenza M.R., Krämer A. (2016). Centrosome amplification, chromosomal instability and cancer: Mechanistic, clinical and therapeutic issues. Chromosome Res..

[42] Nigg E.A., Holland A.J. (2018). Once and only once: Mechanisms of centriole duplication and their deregulation in disease. Nat. Rev. Mol. Cell Biol..

[43] Godinho S.A., Picone R., Burute M., Dagher R., Su Y., Leung C.T., Polyak K., Brugge J.S., Théry M., Pellman D. (2014). Oncogene-like induction of cellular invasion from centrosome amplification. Nature.

[44] Kleylein-Sohn J., Westendorf J., Le Clech M., Habedanck R., Stierhof Y.D., Nigg E.A. (2007). Plk4-induced centriole biogenesis in human cells. Dev. Cell.

[45] Shinmura K., Kurabe N., Goto M., Yamada H., Natsume H., Konno H., Sugimura H. (2014). PLK4 overexpression and its effect on centrosome regulation and chromosome stability in human gastric cancer. Mol. Biol. Rep..

[46] Ling H., Hanashiro K., Luong T.H., Benavides L., Fukasawa K. (2015). Functional relationship among PLK2, PLK4 and ROCK2 to induce centrosome amplification. Cell Cycle.

[47] Hirsch F.R., Suda K., Wiens J., Bunn P.A. (2016). New and emerging targeted treatments in advanced non-small-cell lung cancer. Lancet.

[48] Wang Y., Blandino G., Givol D. (1999). Induced p21waf expression in H1299 cell line promotes cell senescence and protects against cytotoxic effect of radiation and doxorubicin. Oncogene.

[49] Boggaram V. (2009). Thyroid transcription factor-1 (TTF-1/Nkx2.1/TITF1) gene regulation in the lung. Clin. Sci. (Lond.).

[50] Yatabe Y., Mitsudomi T., Takahashi T. (2002). TTF-1 expression in pulmonary adenocarcinomas. Am. J. Surg. Pathol..

[51] Qian H.H., Xu T.S., Cai X.Q., Ji T.L., Guo H.X. (2015). Prognostic value of TTF-1 expression in patients with non-small cell lung cancer: A meta-analysis. Clin. Chim. Acta.

[52] cBioPortal. https://www.cbioportal.org/.

[53] Khozin S., Blumenthal G.M., Jiang X., He K., Boyd K., Murgo A., Justice R., Keegan P., Pazdur R.U.S. (2014). Food and Drug Administration approval summary: Erlotinib for the first-line treatment of metastatic non-small cell lung cancer with epidermal growth factor receptor exon 19 deletions or exon 21 (L858R) substitution mutations. Oncologist.

[54] Piva S., Ganzinelli M., Garassino M.C., Caiola E., Farina G., Broggini M., Marabese M. (2014). Across the universe of K-RAS mutations in non-small-cell-lung cancer. Curr. Pharm. Des..

[55] Nguyen-Ngoc T., Bouchaab H., Adjei A.A., Peters S. (2015). *BRAF* Alterations as therapeutic targets in non-small-cell lung cancer. J. Thorac. Oncol..

[56] Matsuda T., Yagi T., Kawanishi M., Matsui S., Takebe H. (1995). Molecular analysis of mutations induced by 2-chloroacetaldehyde, the ultimate carcinogenic form of vinyl chloride, in human cells using shuttle vectors. Carcinogenesis.

[57] Kawanishi M., Matsuda T., Sasaki G., Yagi T., Matsui S., Takebe H. (1998). A spectrum of mutations induced by crotonaldehyde in shuttle vector plasmids propagated in human cells. Carcinogenesis.

[58] Shinmura K., Goto M., Suzuki M., Tao H., Yamada H., Igarashi H., Matsuura S., Maeda M., Konno H., Matsuda T. (2011). Reduced expression of MUTYH with suppressive activity against mutations caused by 8-hydroxyguanine is a novel predictor of a poor prognosis in human gastric cancer. J. Pathol..

[59] Shinmura K., Kato H., Kawanishi Y., Goto M., Tao H., Yoshimura K., Nakamura S., Misawa K., Sugimura H. (2019). Defective repair capacity of variant proteins of the DNA glycosylase NTHL1 for 5-hydroxyuracil, an oxidation product of cytosine. Free Radic. Biol. Med..

