# Supplementary material for: POLQ Overexpression Is Associated with an Increased Somatic Mutation Load and PLK4 Overexpression in Lung Adenocarcinoma
Source: Cancers (Basel). 2019 May 24;11(5):722. doi: 10.3390/cancers11050722 (PMC6562496; doi:10.3390/cancers11050722)
Supplement: Supplementary file 1 [file cancers-11-00722-s001.pdf]

Supplementary Materials: POLQ Overexpression Is Associated with an Increased Somatic Mutation Load and PLK4 Overexpression in Lung Adenocarcinoma

Kazuya Shinmura, Hisami Kato, Yuichi Kawanishi, Katsuhiro Yoshimura, Kazuo Tsuchiya, Yoshiyuki Takahara, Seiji Hosokawa, Akikazu Kawase, Kazuhito Funai and Haruhiko Sugimura

- Supplementary Table S1: List of translesion DNA synthesis (TLS) polymerase genes analyzed in this study
- Supplementary Table S2: Tumor types from the TCGA database analyzed in this study
- Supplementary Figure S1: Another representative set of results of IHC analysis for concurrent overexpression of PLK4 and POLQ proteins in a case of LAC
- Supplementary Figure S2: Results of the box-plot analyses of cancer types showing increased expression of the TLS polymerase gene other than POLQ associated with an increased number of somatic mutations using data from the TCGA database; the data is linked with Figure 6a
- Supplementary Figure S3: Detection of concurrent overexpression of POLQ and PLK4 in diverse human cancers determined using data from the TCGA database; the data is linked with Figure 6c
- Supplementary Figure S4: Representative images of the staining intensities in the IHC analysis
- Supplementary Figure S5: Blots, molecular markers, and densitometric intensity ratios in Western blot analysis

Table S1. List of translesion DNA synthesis (TLS) polymerase genes analyzed in this study.

TLS gene name	Description	OMIM ID
POLB	DNA Polymerase Beta	174760
POLH	DNA Polymerase Eta	603968
POLI	DNA Polymerase Iota	605252
POLK	DNA Polymerase Kappa	605650
POLL	DNA Polymerase Lambda	606343
POLM	DNA Polymerase Mu	606344
POLN	DNA Polymerase Nu	610887
REV1	REV1, DNA Directed Polymerase	606134
REV3L	REV3 Like, DNA Directed Polymerase Zeta Catalytic Subunit	602776
POLQ	DNA Polymerase Theta	604419

OMIM; Online Mendelian Inheritance in Man.

Table S2. Tumor types from the TCGA database analyzed in this study.

Tumor types	TCGA ID	Number of tumor cases analyzed in Figures 2 and 6
Bladder urothelial carcinoma	BLCA	408
Breast invasive carcinoma	BRCA	1041
Cervical squamous cell carcinoma and endocervical adenocarcinoma	CESC	302
Cholangiocarcinoma	CHOL	36
Colon adenocarcinoma	COAD	284
Head and neck squamous cell carcinoma	HNSC	501
Chromophobe renal cell carcinoma	KICH	66
Clear cell renal cell carcinoma	KIRC	324
Papillary renal cell carcinoma	KIRP	196
Liver hepatocellular carcinoma	LIHC	198
Lung adenocarcinoma	LUAD	513
Lung squamous cell carcinoma	LUSC	492
Pancreatic adenocarcinoma	PAAD	84
Prostate adenocarcinoma	PRAD	495
Rectal adenocarcinoma	READ	89
Stomach adenocarcinoma	STAD	412
Thyroid carcinoma	THCA	486
Uterine corpus endometrial carcinoma	UCEC	159

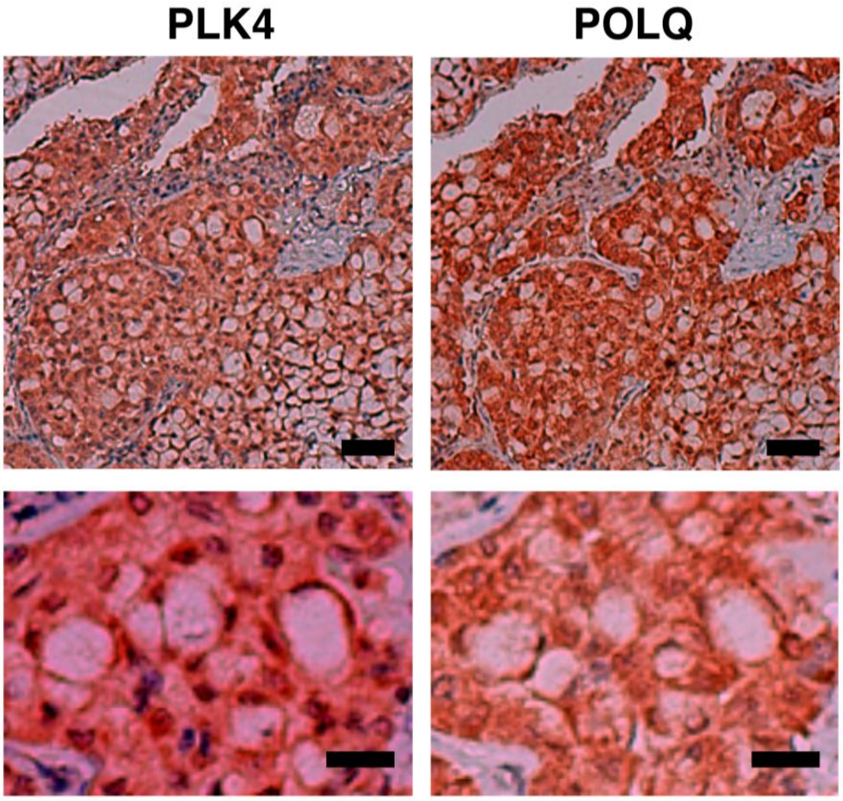

Figure S1. Another representative set of results of IHC analysis for concurrent overexpression of PLK4 and POLQ proteins in a case of LAC. Results of IHC analysis for PLK4 and POLQ proteins in the same LAC. The lower panels show a part of the upper panels at a higher magnification. Scale bar = 50 μm (upper); 20 μm (lower).

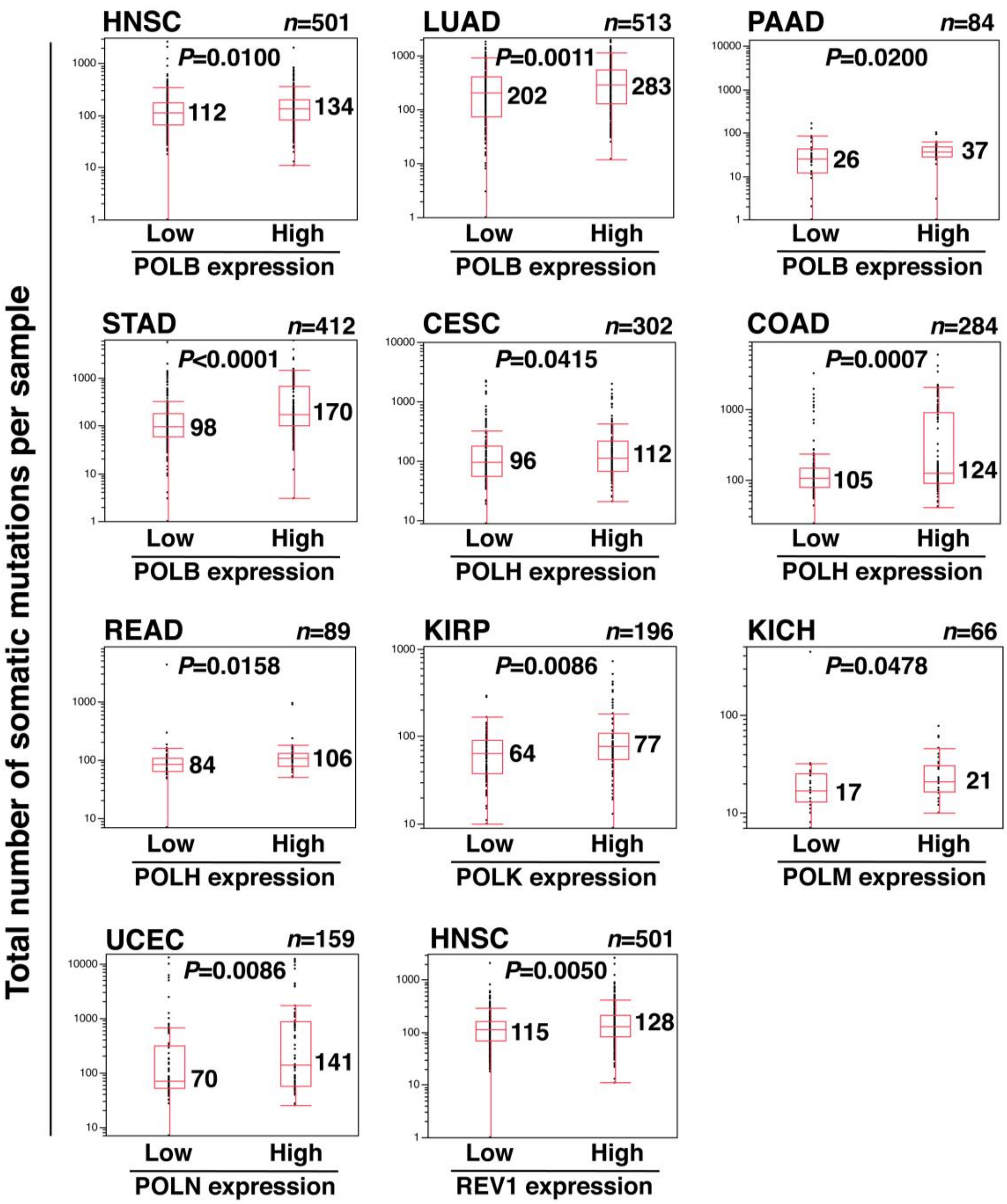

Figure S2. Results of the box-plot analyses of cancer types showing increased expression of the TLS polymerase gene other than POLQ associated with an increased number of somatic mutations using data from the TCGA database; the data is linked with Figure 6a. Median expression value in the cancer tissue samples was used as the cut-off value to dichotomize the cancer cases according to the expression levels of the TLS polymerase genes, and the total numbers of somatic mutations were compared between the cancers showing low TLS polymerase gene expression and those showing high TLS polymerase gene expression using the Mann–Whitney *U* test. The median mutation number in each group and the *p*-values are shown in the graph.

.

Supplementary Figure S3

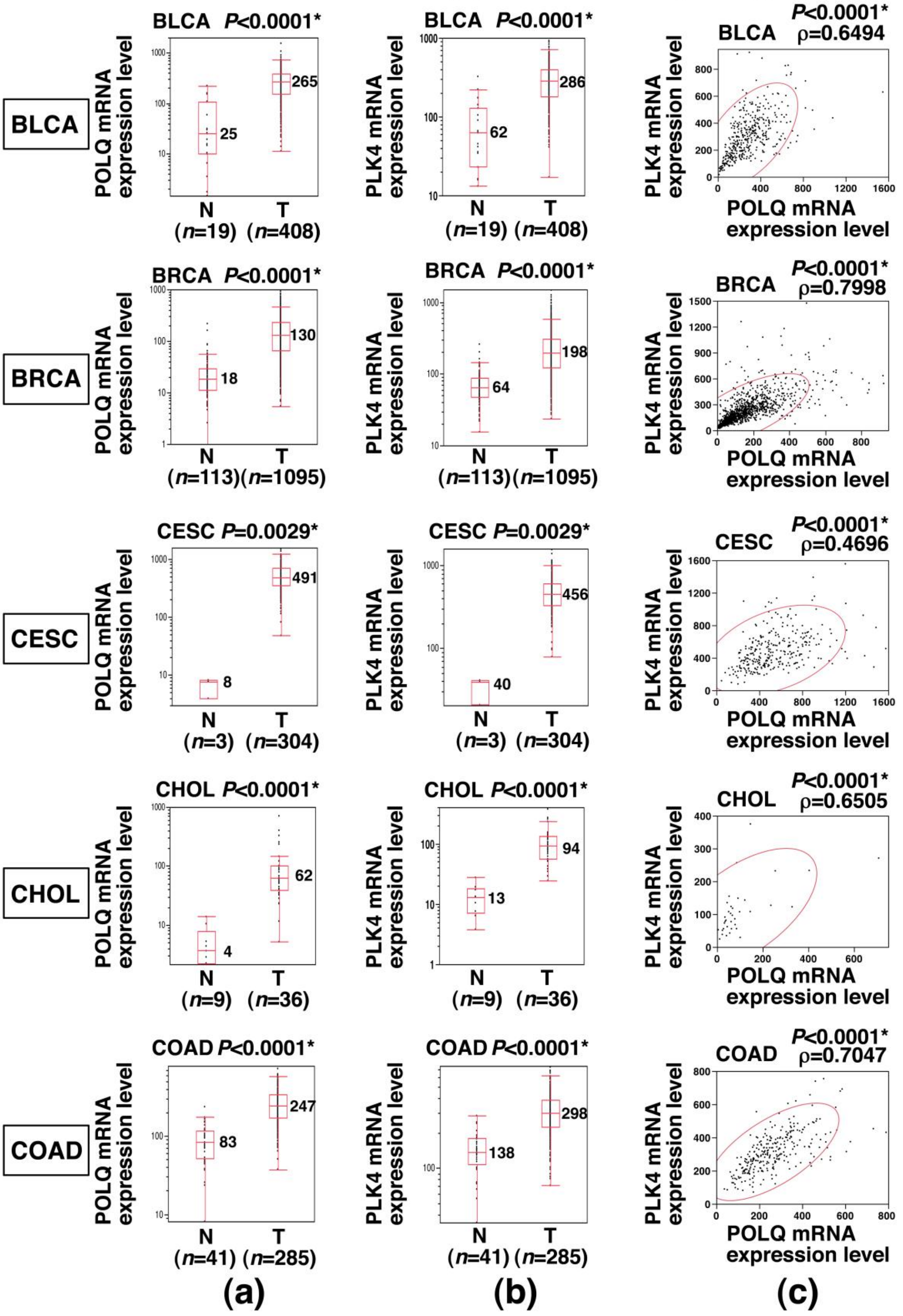

Supplementary Figure S3 (continued)

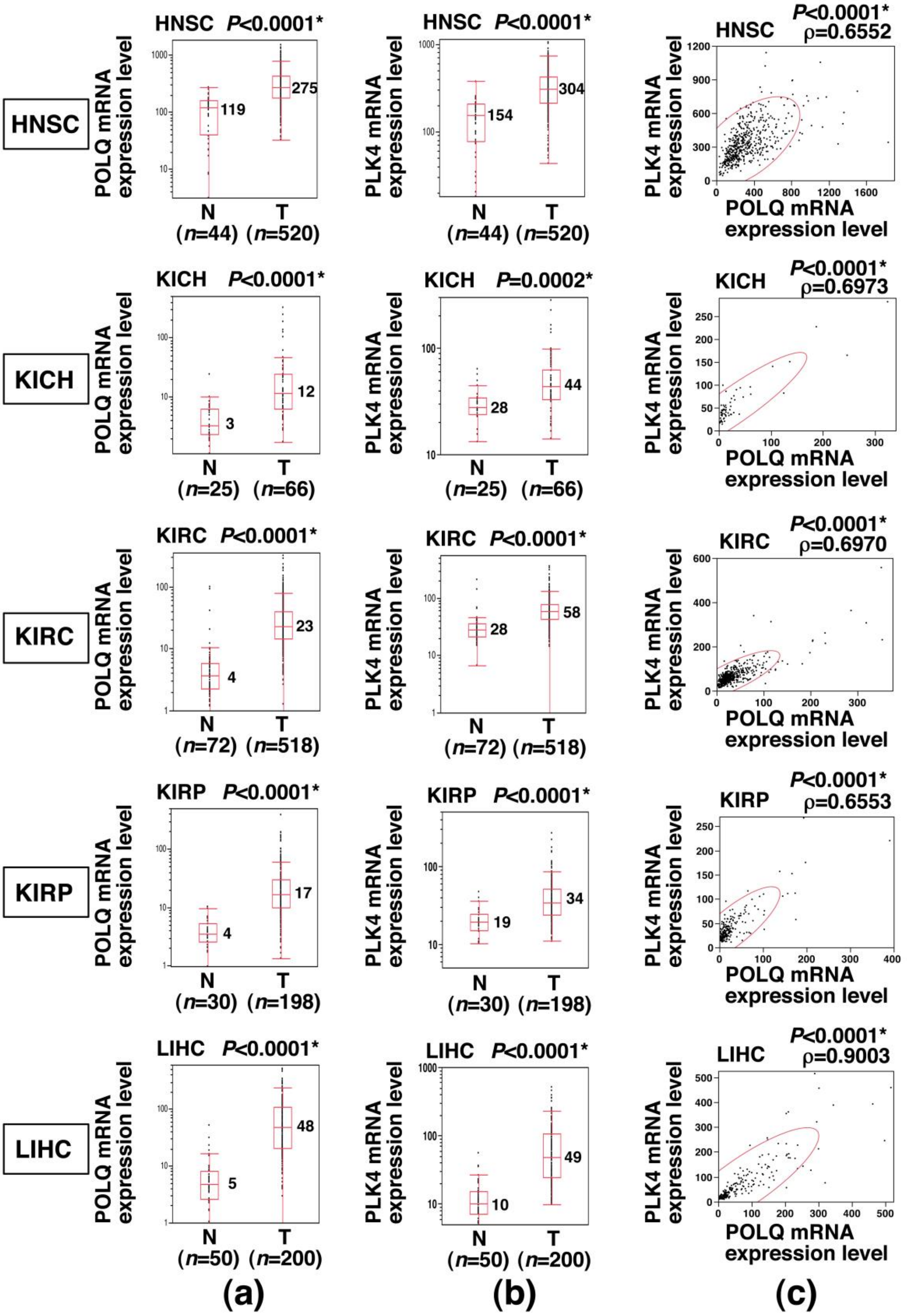

Supplementary Figure S3 (continued)

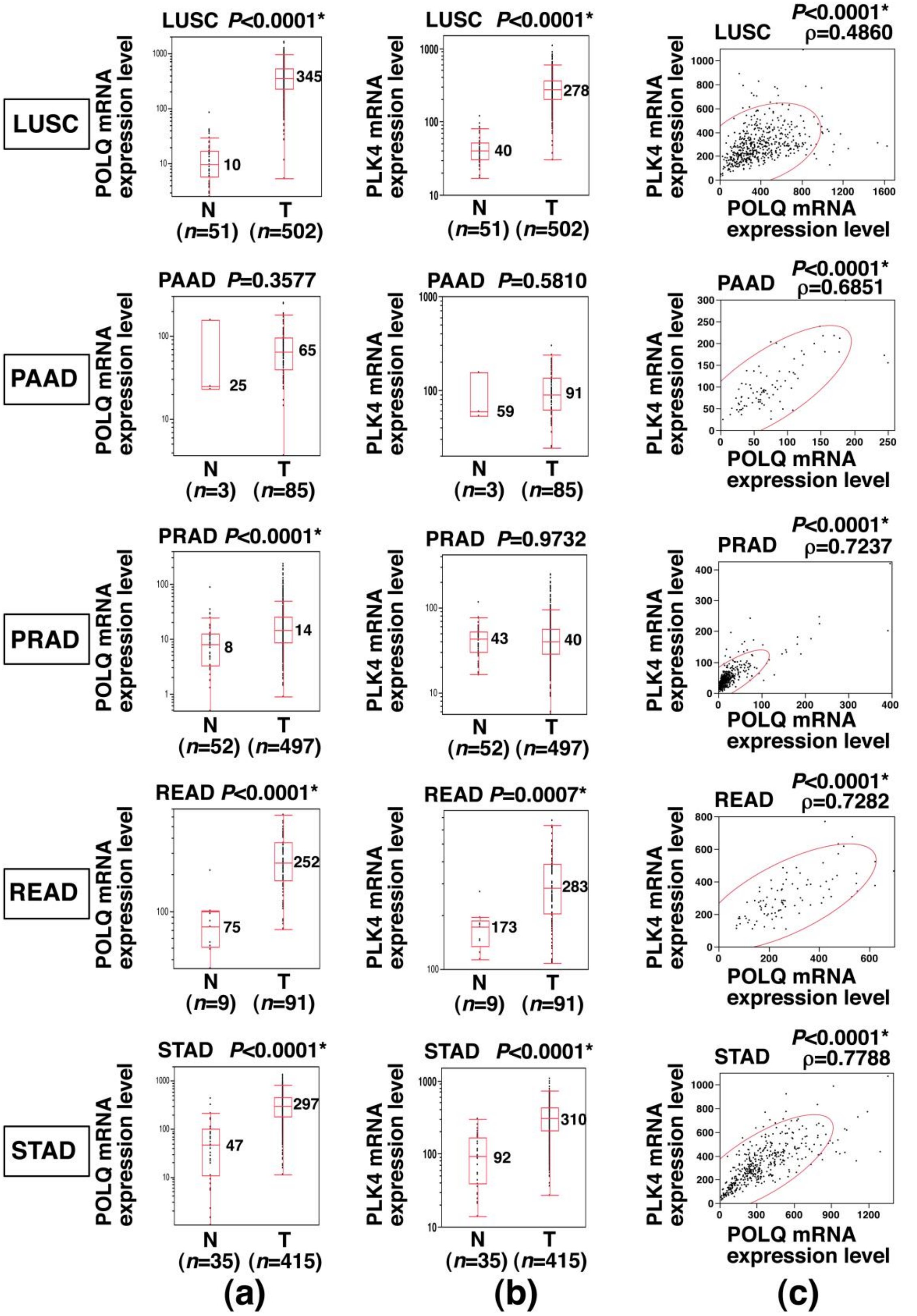

Supplementary Figure S3 (continued)

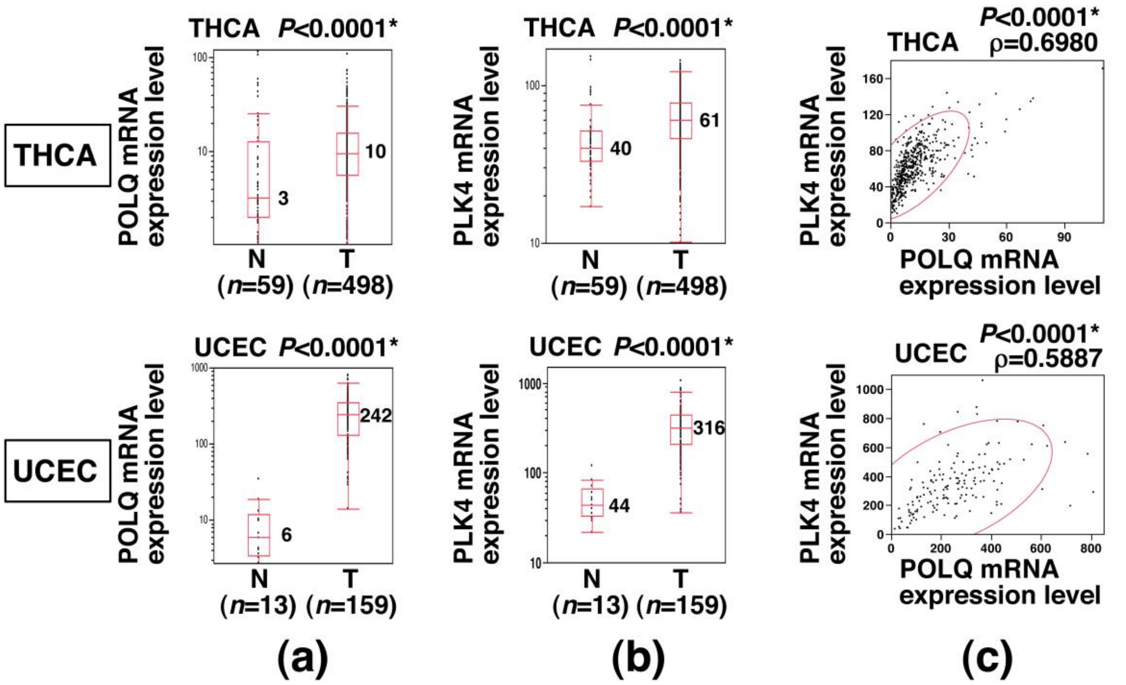

Figure S3. Detection of concurrent overexpression of POLQ and PLK4 in diverse human cancers determined using data from the TCGA database; the data is linked with Figure 6c. (**a**) Detection of POLQ mRNA overexpression in diverse human cancers. A Mann–Whitney *U* test was used for statistical comparison of the findings between non-cancerous tissue (N) and cancerous tissue (T) derived from 17 cancer types; the *p*-values and median expression levels are shown. (**b**) Detection of PLK4 mRNA overexpression in diverse human cancers. A Mann–Whitney *U* test was used for statistical comparison of the findings between non-cancerous tissue (N) and cancerous tissue (T) derived from 17 cancer types; the *p*-values and median expression levels are shown. (**c**) A significant positive correlation was detected between the POLQ and PLK4 mRNA expression levels in diverse human cancers. The Spearman rank correlation coefficient (ρ) and *p*-values are shown. A bivariate normal ellipse ($p = 0.95$) was obtained. The asterisks show a statistically significance in (a)–(c).

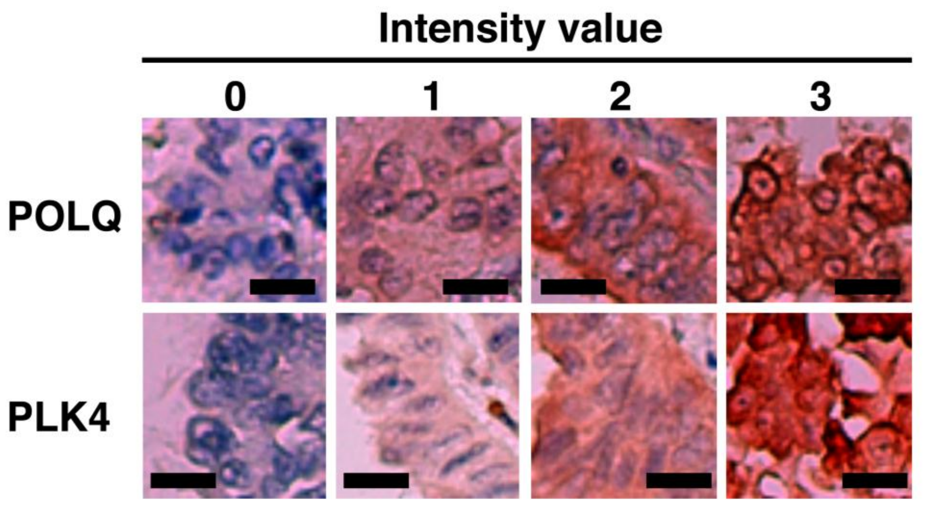

Figure S4. Representative images of the staining intensities in the IHC analysis. Representative images of intensity value (0, absent; 1, weak; 2, moderate; 3, strong) to assess the protein expression level of POLQ and PLK4 are shown. Scale bar = 20 μm.

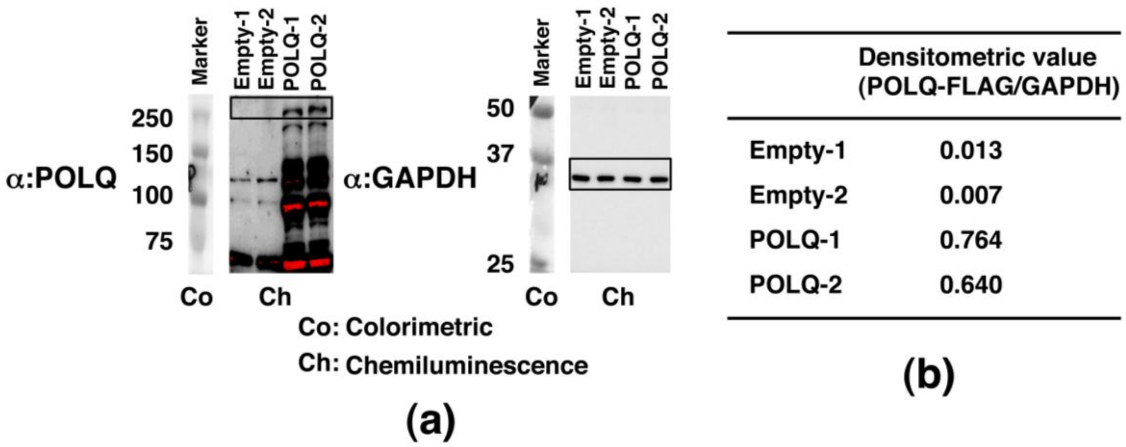

Figure S5. Blots, molecular markers, and densitometric intensity ratios in Western blot analysis. (**a**) The results of chemiluminescence and colorimetric detection for Figure 3a are shown. The rectangle is a photograph of Figure 3a. (**b**) Densitometric intensity ratios (POLQ-FLAG/GAPDH) measured using the ImageJ software (National Institute of Health, Bethesda, MD, USA) are shown.
